# Concordance of the 21-gene assay between core needle biopsy and resection specimens in early breast cancer patients

**DOI:** 10.1007/s10549-020-06075-6

**Published:** 2021-01-13

**Authors:** Peng Qi, Yu Yang, Qian-ming Bai, Tian Xue, Min Ren, Qian-lan Yao, Wen-tao Yang, Xiao-yan Zhou

**Affiliations:** 1 Department of Pathology, Fudan University Shanghai Cancer Center, No. 270 Dong An Road, Shanghai, 200032 China; 2 Department of Oncology, Shanghai Medical College, Fudan University, Shanghai, 200032 China; 3 Institute of Pathology, Fudan University, Shanghai, 200032 China

**Keywords:** Core needle biopsy, Resection specimen, Breast cancer, Predictive biomarkers, Genomic profiling

## Abstract

**Background:**

Adjuvant therapy decisions may be partly based on the results of a multigene quantitative reverse transcription-polymerase chain reaction (RT-PCR)-based assay: the 21-gene recurrence score (RS) test of resection specimens. When necessary, core needle biopsy (CNB) may be considered as a surrogate. Here, we evaluated the concordance in gene expression according to results from RT-PCR-based RS testing between paired CNBs and resection specimens.

**Methods:**

CNBs and resection specimens from 50 breast cancer (BC) patients were tested to calculate RSs. First, we examined the concordance of the ER, PR and HER-2 status of tissue samples indicated by immunohistochemical (IHC) and RT-PCR analyses. Then, we compared the IHC findings of ER, PR, HER-2 and Ki-67 staining across paired samples. Ultimately, the RS and single-gene results for ER, PR, HER-2 and Ki-67 were explored between paired samples.

**Results:**

The concordance between IHC and RT-PCR was 100%, 80.0% and 100% for ER, PR and HER-2, respectively, in both resection specimens and CNBs. The concordance for IHC ER, PR, HER-2 and Ki-67 status was 100%, 94.0%, 52.0% and 82.0%, respectively, between paired samples. RS results from paired samples showed a strong correlation. The overall concordance in RS group classification between samples was 74%, 72% and 78% based on traditional cutoffs, TAILORx cutoffs and ASCO guidelines, respectively. ER, PR, HER-2 and Ki-67 were modestly- to- strongly correlated between paired samples according to the RT-PCR results.

**Conclusion:**

A modest- to- strong correlation of ER, PR, HER-2 and Ki-67 gene expression and RS between CNBs and resection specimens was observed in the present study. The 21-gene RS test could be reliably performed on CNBs. ER, PR and HER-2 status showed remarkable concordance between the IHC and RT-PCR analyses. The concordance between paired samples was high for the IHC ER, PR and Ki-67 results and low for HER-2.

## Introduction

Breast cancer (BC) is the leading cause of death in women aged 35–55 and the second leading cause of death among women of all ages [1]. Optimal systemic treatment (adjuvant therapy) after surgery is a crucial factor in reducing mortality in BC patients [2]. The decisions surrounding the systemic treatment of BC have traditionally been based on a combination of clinical and histopathologic risk factors, including tumour size [3], histologic grade [4], measures of proliferation [5] and lymph node status [6]. In addition, the oestrogen receptor (ER), progesterone receptor (PR) and human epidermal growth factor receptor-2 (HER-2) proteins are crucial biomarkers used to classify BCs into subgroups and subsequently guide adjuvant treatment decisions, including antioestrogen or targeted therapy, in the management of BC [7–9]. These prognostic markers are highly useful for identifying higher-risk triple-negative, potential endocrine therapy benefit or HER-2-positive cases; however, they appear to present limited predictive value for assessing responsiveness to chemotherapy in the lower-risk subset of hormone receptor (HR)-positive patients on a case-by-case basis.

Over the last decade, various multigene assays for predicting prognosis and treatment response have entered the clinical arena of BC management [10, 11]. The 21-gene assay (Oncotype DX) is one such test. It is a quantitative reverse transcription-polymerase chain reaction (RT-PCR)-based assay that analyses 16 cancer-related and 5 reference genes to provide a recurrence score (RS) using RNA extracted from formalin-fixed paraffin-embedded (FFPE) tumour tissues. RS is reported as a number ranging from 0 to 100 and is divided into low, intermediate, and high recurrence risk categories. The 21-gene RS test has been shown to be prognostic [12] and predictive of chemotherapy benefit [13] in early-stage, HR+, lymph node-negative, or lymph node-positive BC.

The 21-gene assay can be applied regularly to resection specimens or to core needle biopsies (CNBs) [14]. For years, BC resection specimens have been considered the gold standard for obtaining information from immunohistochemistry (IHC) tests or other molecular tests because they exhibit the most abundant tumour characteristics. However, CNBs have become increasingly important in the preoperative work-up of BC patients, especially in those who receive neoadjuvant treatment. The use of CNBs for the determination of RS has the advantage of making the final result available before the surgical procedure. Second, CNBs are the only material available from patients under neoadjuvant treatment. Another advantage of CNBs is the more standardized fixation protocols applied to these specimens. For resection specimens, many different protocols are available, and the time from the interruption of the blood supply to the initiation of fixation is likely longer, since surgery is a more complicated procedure than CNB. It is important to adhere to good laboratory practices regarding tissue handling and fixation [15]. However, at some centres, breast resection specimens are not immediately sliced and fixed, resulting in poor fixation of the tissue. Additionally, preanalytical factors can interfere with accurate immunohistochemical scoring and molecular testing. For example, Mann et al. [16] concluded that HR analysis of CNBs appears to be more reliable than the analysis of resection specimens, probably due to the loss of antigens in resection specimens caused by the fixation process. However, a disadvantage of using CNB is the possibility of crush artefacts, which may lead to inaccurate results owing to tumour heterogeneity. Richter-Ehrenstein et al. [17] stated that CNBs were less accurate than resection specimens for determining the histological characteristics of a tumour, such as tumour invasiveness and tumour grade, particularly in larger and/or heterogeneous tumours. Gerlinger et al. [18] also noted that intratumoural heterogeneity can lead to the underestimation of tumour genomic characteristics owing to the varying gene expression signatures in different regions of the same tumour.

Ideally, the 21-gene assay should accurately represent actual tumour characteristics if these characteristics will guide treatment. Although several sets of guidelines recommend this assay for HR + , lymph node-negative, early-stage BC [19–22], it has not been compared head-to-head between CNBs and resection specimens, and little data are available regarding the correlation of RS in these specimen types. Previously, several studies have demonstrated concordant and discordant IHC findings regarding the ER, PR, HER-2 and Ki-67 status of breast lesions between CNBs and resection specimens [23–25]. Patients with false-negative test results may be undertreated, whereas patients with false-positive results may receive unnecessary treatment with expensive drugs, potentially causing serious side effects. These potential risks emphasize the importance of proper diagnostic procedures for the determination of ER, PR, HER-2 and Ki-67 status, similar to the 21-gene assay. Quantitative single-gene results for ER, PR and HER-2 are assessed and reported as part of the RS assay. These three genes are routinely measured at the protein level by IHC. Although the RT-PCR results have been shown to be highly concordant with the results of IHC/fluorescent in situ hybridization (FISH) assays [26, 27], the potential variability in RS values between CNBs and resection specimens is multifactorial and can be affected by single-gene expression, tumour heterogeneity and pre-analytic differences such as the delay to fixation, duration of fixation, or differences in fixatives. Furthermore, it has been reported that the presence of increased stromal cellularity and/or associated inflammatory cells and tumour-infiltrating lymphocytes (TILs) may contribute to an apparently increased recurrence risk according to the 21-gene assay [28, 29]. Consequently, in this study, we assessed the ER, PR, HER-2 and Ki-67 status and RS value in paired CNBs and resection specimens from 50 BC patients. The goals of the present study were (1) to assess the correlation of RS, ER, PR, HER-2 and Ki-67 values determined by RT-PCR [12, 30] between paired resection specimens and CNB samples; (2) to investigate the relationship between IHC and RT-PCR assessments of ER, PR and HER-2 in tissue samples; and (3) to compare the ER, PR, HER-2 and Ki-67 status between paired samples by IHC. The observation of a high degree of concordance between these tumour tissues may be clinically valuable for untreated patients presenting with CNBs and resection specimens. If so, then molecular assays that have been developed previously and validated using only resection specimens might also be applied to CNBs, and if not, the results might reveal whether CNB analyses tend to over- or underestimate tumour activity.

## Methods

### Patient selection and clinicopathological data

We selected 50 consecutive patients with early-stage, ER-positive, HER-2-negative, lymph node-negative invasive breast carcinoma who were subjected to the 21-gene assay as part of their clinical care between 1 May 2020 and 31 July 2020. All patients were > 18 years of age without a prior history of any cancer, including BC. All haematoxylin and eosin (H&E)-stained slides of their resection specimens were reviewed to establish the diagnosis, including the histologic type and grade [31, 32], and the cancers were grouped as follows: invasive ductal carcinoma (IDC), invasive lobular carcinoma (ILC), mucinous carcinoma (MC) or mixed-type invasive carcinoma as the histologic type; grade 1 (G1), grade 2 (G2) or grade 3 (G3) as the tumour grade. Patients were subdivided into luminal A-like and luminal B-like subtypes according to the 2013 St Gallen International Expert Consensus [33]. The tumour stage was obtained according to the American Joint Committee on Cancer (AJCC) TNM staging system [34].

The determination of ER, PR, HER-2 and Ki-67 status was routinely carried out in the Department of Pathology, Fudan University Shanghai Cancer Center, for both FFPE resection specimens and preoperative CNBs. Antibodies against the following proteins were used for the IHC test: ER: clone SP1 (rabbit monoclonal, Roche), PR: clone 1E2 (rabbit monoclonal, Roche), HER-2: clone 4B5 (rabbit monoclonal, Roche), and Ki-67: clone 30–9 (rabbit monoclonal, Roche). The IHC slides were assessed as part of the daily routine of two pathologists, one of whom was an experienced breast pathology consultant. The cutoff point for ER and PR positivity was set at 1% [35]. HER-2 overexpression was scored as 1 + (incomplete membrane staining of any proportion of tumour cells), 2+ (complete membrane staining that was either nonuniform or weak in intensity but showed an obvious circumferential distribution in at least 10% of tumour cells, or invasive tumours with intense, complete membrane staining of not more than 10% of tumour cells), or 3 + (uniform, intense membrane staining of 10% of invasive tumour cells), in accordance with the American Society of Clinical Oncology (ASCO)/CAP guidelines [36]. Tumours with IHC scores of 0 or 1 + were defined as HER-2-negative. Tumours with a 2 + score were subjected to a FISH assay using the PathVysion DNA Probe Kit (Abbott Molecular, Abbott Park, IL), a Duet scanning imaging workstation (BioView, Billerica, MA), and the accompanying software. A positive result was defined according to the updated recommendations [37]. For Ki-67 assessments, a cutoff point of 20% was used in reference to the 2013 St Gallen International Expert Consensus [33].

### RNA extraction, amplification, and gene expression analysis by RT-PCR

RS was determined from FFPE tissue as previously described [12]. In brief, H&E slides were reviewed to ensure the presence of sufficient invasive BC, and RNA was then extracted from eight 5-μm unstained sections. The total RNA content was measured, and the absence of DNA contamination was verified. Gene-specific reverse transcription was performed, followed by standardized quantitative RT-PCR in 384-well plates using the Applied Biosystems QuantStudio™ Dx Real-Time PCR System (Foster City, CA). The expression of each gene was measured and normalized relative to a set of five reference genes. The reference-normalized expression measurements range from 0 to 15, and a 1-unit increase reflects an approximately twofold increase in RNA expression. A tumour is ER-negative when it presents < 6.5 expression units; ER-positive, ≥ 6.5; PR-negative, < 5.5; PR-positive, ≥ 5.5; HER-2-negative, < 10.7; HER-2-equivocal, ≥ 10.7–11.4; or HER-2-positive, ≥ 11.5 [38]. RS, ranging from 0 to 100, was derived from the reference-normalized expression measurements for the 16 cancer-related genes. Patients were then categorized into low-risk (< 18), intermediate-risk (18–30), and high-risk (≥ 31) groups [12]. We also investigated low- and midrange-risk groups as follows: low (< 11), intermediate (11–25), and high (≥ 26) [39–41].

### Statistical analysis

Patient characteristics were summarized using descriptive statistics. Analyses were performed on all patients with reportable results from the 21-gene assay for both the resection specimens and CNBs. Scatterplots of the ER, PR, HER-2 and Ki-67 expression values and RS results from the resection specimens and CNBs were constructed. The chi-square test was applied to evaluate the distribution of RS risk categories among patients with different clinicopathological factors. The degree of concordance between the IHC and RT-PCR results with respect to ER, PR and HER-2 status was assessed graphically and according to the kappa statistic. The chi-square test and kappa statistic were applied to compare the IHC findings of ER, PR, HER-2 and Ki-67 staining across paired resection specimens and CNB samples. Pearson’s correlation coefficient and 95% CIs were calculated to compare single-gene ER, PR, HER-2 and Ki-67 expression and RS results as continuous variables between paired samples. Discordance in RS groups was also measured, with one-level discordance defined as low ↔ intermediate or intermediate ↔ high and two-level discordance defined as low ↔ high. The expression of each of the ER, PR and HER-2 genes and the RS correlations between paired samples were determined as categorical variables using the chi-square test. *p* < 0.05 was considered significant. All statistical tests were performed using the statistical package SPSS (version 16.0 for Windows, SPPS, Inc., Chicago, IL, USA).

## Results

### Patient characteristics

The clinicopathological characteristics of the 50 included patients who were treated at our hospital are summarized in Table 1. These patients were all treated for early-stage BC with primary surgery. Their mean age was 51 (range: 29–72 years). Mastectomy was performed in 32 (64%) patients. Based on the resection specimens, the most common histological subtype was IDC, which was diagnosed in 44 patients (88%). Grade 1, 2, and 3 tumours were documented in 2%, 92% and 6% of the patients, respectively. The median tumour size was 2.0 cm (range: 0.4–4.5 cm). T1 tumours were found in 54% of all patients. The majority of patients were identified as PR-positive (92%) and presented a low Ki-67 index (66%).

**Table 1:** Patient and tumour characteristics

	*N*	%
Age		
≤ 50	27	54
> 50	23	46
Surgery		
Mastectomy	32	64
BCS	18	36
Pathology		
IDC	44	88
ILC	2	4
MC	2	4
Invasive carcinoma, mixed	2	4
Grade		
I	1	2
II	46	92
III	3	6
T stage		
T1	27	54
T2	23	46
ER		
Positive	50	100
Negative	0	0
PR (resection specimen)		
Positive	46	92
Negative	4	8
PR (CNB)		
Positive	47	94
Negative	3	6
HER-2 (resection specimen)		
0	9	18
1+	32	64
2+	9	18
HER-2 (CNB)		
0	7	14
1+	30	60
2+	13	26
Ki-67 (resection specimen)		
< 20	33	66
≥ 20	17	34
Ki-67 (CNB)		
< 20	30	60
≥ 20	20	40
Subtype		
Luminal A-like	32	64
Luminal B-like	18	36
RS category (resection specimen)		
Low-risk (< 18)	12	24
Intermediate-risk (18–30)	33	66
High-risk (≥ 31)	5	10
RS category (resection specimen)		
Low-risk (< 11)	1	2
Intermediate-risk (11–25)	30	60
High-risk (≥ 26)	19	38
RS category (CNB)		
Low-risk (< 18)	13	26
Intermediate-risk (18–30)	26	52
High-risk (≥ 31)	11	22
RS category (CNB)		
Low-risk (< 11)	3	6
Intermediate-risk (11–25)	26	52
High-risk (≥ 26)	21	42

*BCS* breast conserving surgery; *IDC* invasive ductal carcinoma; *ILC* invasive lobular carcinoma; *MC* mucinous carcinoma; *ER* oestrogen receptor; *PR* progesterone receptor; *HER-2* human epidermal growth factor receptor-2; *CNB* core needle biopsy; *RS* recurrence score

### Distribution of RS

**Fig. 1:**
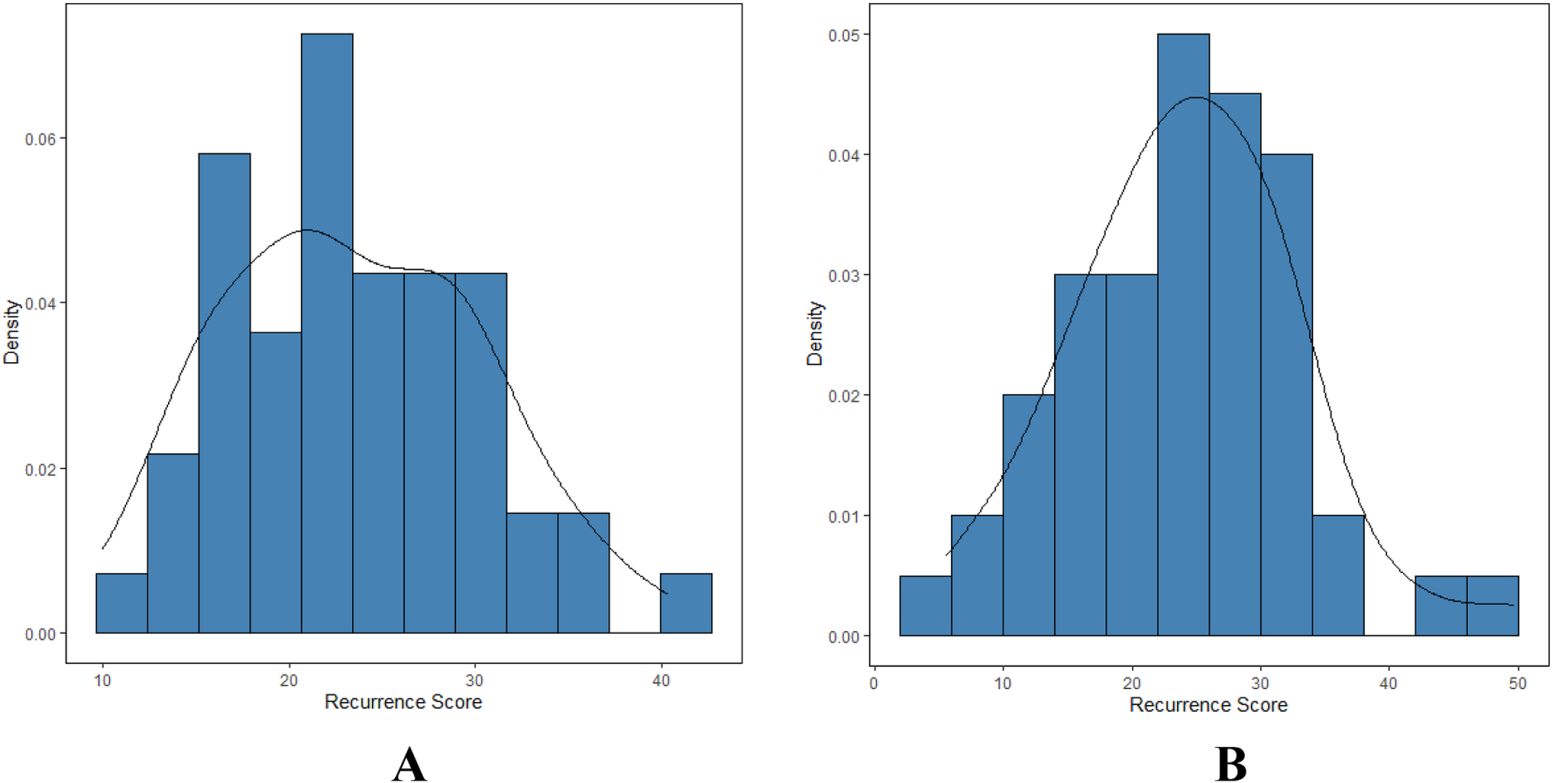
Recurrence score distribution in 50 patients. **a** Resection specimen. **b** Core needle biopsy (CNB)

Among the 50 patients, the mean RS for the resection specimens was 23.4 (range: 10–40), compared to 24.2 (range: 5–50) for CNBs, and RS followed a normal distribution (Fig. 1). According to the RS results of resection specimens, 12 patients were (24%) in the low-risk group (RS < 18), 33 patients (66%) were in the intermediate-risk group (RS 18–30), and 5 patients (10%) were in the high-risk group (RS ≥ 31). The distribution of RS varied significantly according to different T stages, PR statuses and molecular subtypes (all *p* < 0.05, Table 2). According to the TAILORx RS cutoffs and the RS results of the resection specimens, 1 patient (2%) was in the low-risk group (RS < 11), 30 patients (60%) were in the intermediate-risk group (RS 11–25), and 19 patients (38%) were in the high-risk group (RS ≥ 26). The distribution of RS varied significantly according to age, PR status and molecular subtypes (all *p* < 0.05, Table 2). Negative PR and luminal B subtypes were more likely to present high-risk RSs.

**Table 2:** Distribution of RS according to clinicopathologic factors

Characteristics	RS category, *N* (resection specimen) by traditional cutoffs			*p*	RS category, *N* (resection specimen) by TAILORx cutoffs			*p*
	Low-risk (< 18)	Intermediate-risk (18–30)	High-risk (≥ 31)		Low-risk (< 11)	Intermediate-risk (11–25)	High-risk (≥ 26)	
Age								
≤ 50	6	20	1	0.212	1	20	6	0.029
> 50	6	13	4		0	10	13	
Surgery								
Mastectomy	5	23	4	0.169	0	17	15	0.094
BCS	7	10	1		1	13	4	
Pathology								
IDC	11	28	5	0.420	1	26	17	0.841
Others	1	5	0		0	4	2	
Grade								
I–II	12	31	4	0.249	1	30	16	0.134
III	0	2	1		0	0	3	
T stage								
T1	3	23	1	0.007	0	16	11	0.433
T2	9	10	4		1	14	8	
PR								
Positive	12	31	3	0.048	1	30	15	0.016
Negative	0	2	2		0	0	4	
HER-2								
0	2	7	0	0.686	0	5	4	0.888
1+	8	20	4		1	19	12	
2+	2	6	1		0	6	3	
Ki-67								
≤ 20	9	23	1	0.077	1	23	9	0.074
> 20	3	10	4		0	7	10	
Subtype								
Luminal like	12	19	1	< 0.001	1	27	4	< 0.001
Luminal like	0	14	4		0	3	15	

*BCS* breast conserving surgery; *IDC* invasive ductal carcinoma; *PR* progesterone receptor; *HER-2* human epidermal growth factor receptor-2; *RS* recurrence score

### Concordance of ER, PR, and HER-2 results between IHC/FISH and RT-PCR

**Fig. 2:**
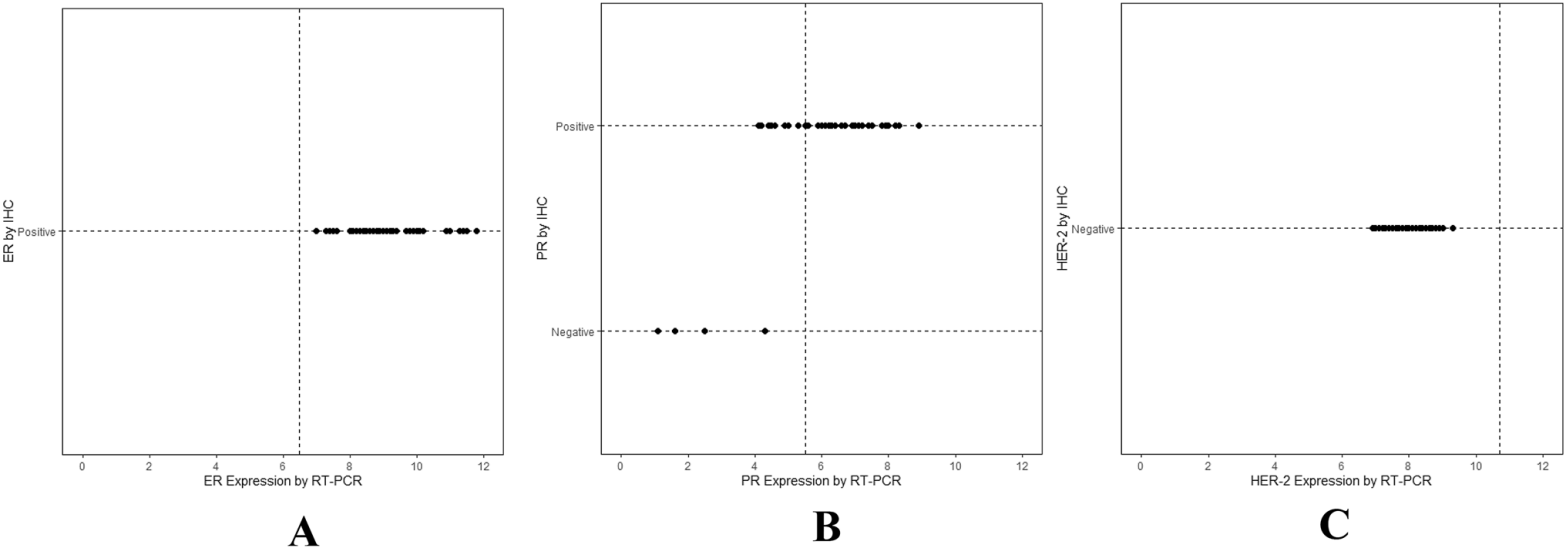
Agreement between IHC/FISH measurements and RT-PCR results for ER, PR and HER-2 in resection specimens. The cutoff points of 6.5, 5.5 and 11.5 were prespecified based on prior studies of the concordance of IHC and RT-PCR

We examined the agreement between the IHC and RT-PCR results for ER, PR and HER-2 using the following prespecified thresholds for RT-PCR gene expression: ER (≥ 6.5), PR (≥ 5.5) and HER-2 (≥ 11.5). Among the resection specimens with a usable IHC/FISH result, 100% were ER-positive, 92% were PR-positive, and 0% were HER-2-positive. According to the prespecified thresholds for ER, PR, and HER-2 expression, 100%, 72.0%, and 0% of these samples, respectively, were positive according to the RT-PCR assay. Figure 2a, c present the expression levels of ER and HER-2 for every sample after stratification according to the IHC/FISH results, and complete agreement between the two assays is demonstrated. Figure 2b shows the concordance between the two assays for PR. For this biomarker, there were 10 samples that were positive by IHC but negative by RT-PCR, whereas there were no samples that were positive by RT-PCR but negative by IHC/FISH. The IHC/FISH and RT-PCR analyses of ER, PR and HER-2 were concordant in 100%, 80% and 100% of the resection specimens, respectively. The kappa coefficient and 95% CI for ER, PR and HER-2 were 1.00 (1.00, 1.00), 0.37 (0.09, 0.64) and 1.00 (1.00, 1.00), respectively, reflecting perfect agreement for ER and HER-2 between the two assays and moderate agreement for PR in the resection specimens (Table 3).

**Table 3:** Concordance of ER, PR and HER-2 status between IHC/FISH and RT-PCR in resection specimen and CNB

Resection specimen					CNB				
IHC/FISH	RT-PCR		Kappa coefficient and 95% CI	Discordant cases (*N*)	IHC/FISH	RT-PCR		Kappa coefficient and 95% CI	Discordant cases (*N*)
ER	Positive	Negative			ER	Positive	Negative		
Positive	50	0	1.00 (1.00, 1.00)	0	Positive	50	0	1.00 (1.00, 1.00)	0
Negative	0	0			Negative	0	0		
PR					PR				
Positive	36	10	0.37 (0.09, 0.64)	10	Positive	37	10	0.31 (0.03, 0.58)	10
Negative	0	4			Negative	0	3		
HER-2					HER-2				
Positive	0	0	1.00 (1.00, 1.00)	0	Positive	0	0	1.00 (1.00, 1.00)	0
Negative	0	50			Negative	0	50		

*ER* oestrogen receptor; *PR* progesterone receptor; *HER-2* human epidermal growth factor receptor-2; *IHC* immunohistochemistry; *FISH* fluorescent in situ hybridization; *CNB* core needle biopsy; CI confidence interval

**Fig. 3:**
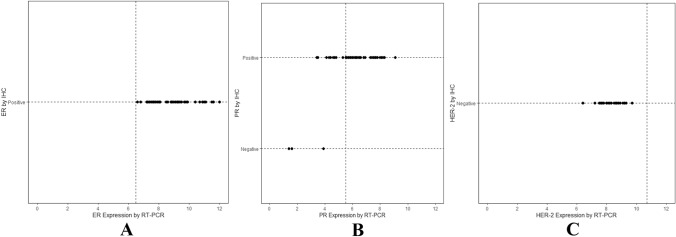
Agreement between IHC/FISH measurements and RT-PCR results for ER, PR and HER-2 in core needle biopsies (CNBs). The cutoff points of 6.5, 5.5 and 11.5 were prespecified based on prior studies of the concordance of IHC and RT-PCR

Among the CNBs with a usable IHC/FISH result, 100% were ER-positive, 94% were PR-positive, and 0% were HER-2-positive. According to the prespecified thresholds for ER, PR and HER-2 expression, 100%, 74% and 0% of these samples, respectively, were positive according to the RT-PCR assay. Figure 3a, c presents the expression levels of ER and HER-2 for every sample after stratification according to the IHC/FISH results and demonstrate complete agreement between the two assays. Figure 3b shows the concordance for PR between the two assays. For this biomarker, there were 10 samples that were positive by IHC but negative by RT-PCR, and there were still no samples that were positive by RT-PCR but negative by IHC. The IHC/FISH and RT-PCR results for ER, PR and HER-2 were concordant in 100%, 80% and 100% of the CNBs, respectively. The kappa coefficient and 95% CI for ER, PR and HER-2 were 1.00 (1.00, 1.00), 0.31 (0.03, 0.58) and 1.00 (1.00, 1.00), respectively, reflecting perfect agreement for ER and HER-2 between the two assays and moderate agreement for PR in CNBs (Table 3).

### Concordance of ER, PR, HER-2 and Ki-67 status by IHC/FISH across paired resection specimens and CNBs

Both the resection specimen and CNB IHC/FISH ER, PR, HER-2 and Ki-67 results were available for all 50 patients. The concordance of separate groups is shown in Table 4. All the patients had tumours that were positive for ER receptor expression in the resection specimen. The final ER results from the CNBs were the same, indicating perfect agreement.

**Table 4:** Concordance for ER, PR, HER-2, and Ki-67 status by IHC/FISH between paired resection specimen and CNB

Resection specimen	CNB			Kappa coefficient and 95% CI	Discordant cases (*N*)	*p*
ER	Positive	Negative				
Positive	50	0		1.00 (1.00, 1.00)	0	
Negative	0	0				
PR	Positive	Negative				
Positive	45	1		0.54 (0.08, 1.00)	3	0.006
Negative	2	2				
HER-2	0	1 +	2 +			
0	4	5	0	0.086 (− 0.09, 0.27)	24	0.017
1+	3	19	10			
2+	0	6	3			
HER-2	Positive	Negative				
Positive	0	0		1.00 (1.00, 1.00)	0	
Negative	0	50				
Ki-67	< 20	≥ 20				
< 20	27	6		0.62 (0.39, 0.84)	9	< 0.001
≥ 20	3	14				

*ER* oestrogen receptor; *PR* progesterone receptor; *HER-2* human epidermal growth factor receptor-2; *IHC* immunohistochemistry; *FISH* fluorescent in situ hybridization; *CNB* core needle biopsy; *CI* confidence interval

Forty-six patients (92%) had tumours that were positive for PR receptor expression in the resection specimen, and 4 (8%) had tumours that were negative for PR expression. The final PR results from the CNBs were positive for 47 tumours (94%) and negative for 3 (6%) tumours. There was a discrepancy in 3 cases, and the concordance was 94% (*p* = 0.006). Two of the discrepant cases showed weak PR positivity (2% of cells) in the CNB specimens and PR negativity in the resection specimens, while 1 case showed PR negativity in the CNB specimen but weak PR positivity (< 10% of cells) in the resection specimen. The kappa coefficient and corresponding 95% CI were 0.54 (0.08, 1.00), indicating moderate agreement (Table 4).

Among the resection specimens, 9 tumours were scored as 0, 32 as 1 + and 9 as 2 + . According to the CNB HER-2 results, 7 tumours were scored as 0, 30 as 1 + and 13 as 2 + . When the results between paired samples were compared using the three IHC scores (0, 1+, 2+), we observed a significant number of discordant results (*p* = 0.017). A total of 24 tumours showed discordance for these three scores, and 26 showed concordance (52%). This resulted in a kappa coefficient of 0.086, indicating poor agreement (Table 4). However, these concordances did not all have clinical implications. Tumours with 2 + scores were subjected to the FISH assay. All 22 of these tumours were HER-2-negative. We then assessed HER-2 status as a dichotomous variable (HER-2-negative/HER-2-positive). The concordance was 100% among all tumours (Table 4).

The Ki-67 proliferation index was < 20% in 66% of resection specimens, < 20% in 60% of CNBs, ≥ 20% in 34% of resection specimens, and ≥ 20% in 40% of CNBs. There were a total of 9 discrepant cases, and the overall concordance was 82% (*p* < 0.001). Six of the discrepant cases showed high Ki-67 proliferation in CNBs and low Ki-67 proliferation in the resection specimens, while 3 cases showed low Ki-67 proliferation in CNBs and high Ki-67 proliferation in resection specimens. The kappa coefficient was 0.62, indicating moderate agreement (Table 4).

### Correlation of RS, ER, PR, HER-2 and Ki-67 values determined by RT-PCR between paired resection specimens and CNB samples

**Fig. 4:**
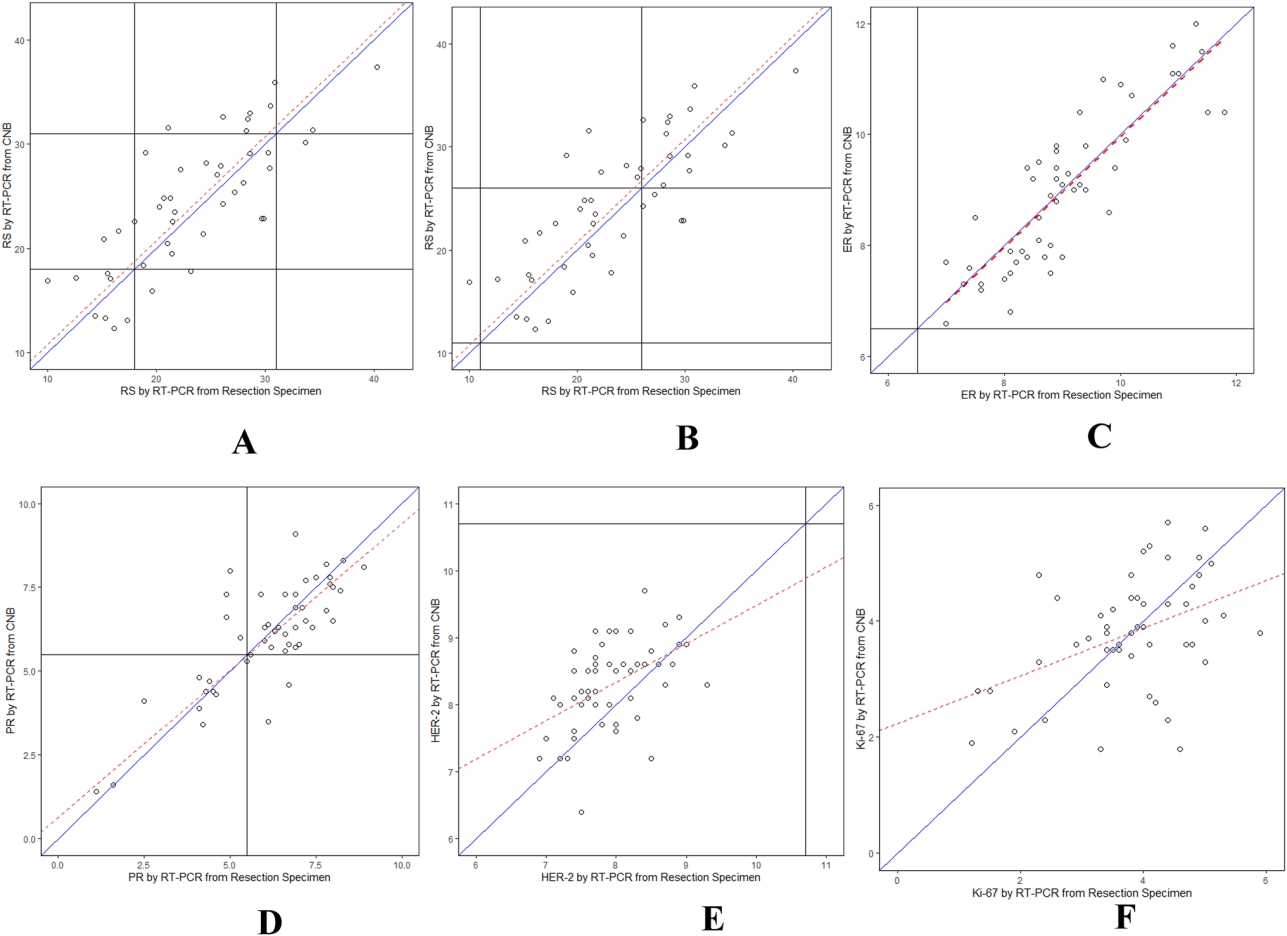
Comparison of gene expression results measured in resection specimens and core needle biopsies (CNBs). Scatterplots of A. RS results using the traditional RS cutoffs; B. RS results using the TAILORx RS cutoffs; C. ER quantification by RT-PCR; D. PR quantification by RT-PCR; E. HER-2 quantification by RT-PCR; F. Ki-67 quantification by RT-PCR in the resection specimens and CNBs

There was a wide distribution of RS results among both the resection specimens and CNB samples. As a continuous variable, RS for resection specimens significantly correlated with RS for CNB (Fig. 4a, b, Pearson’s correlation, 0.80 [95% CI 0.65–0.94]), despite the different definitions of risk categories. Table 5 shows the categorical distribution of RS among the paired resection specimens and CNB samples. The 21-gene assay demonstrated a low RS (< 18) in 12 (24%), intermediate RS (18–30) in 33 (66%) and high RS (≥ 31) in 5 (10%) resection specimens, while it demonstrated a low RS (< 18) in 13 (26%), intermediate RS (18–30) in 26 (52%) and high RS (≥ 31) in 11 (22%) CNBs. When analysed as categorical variables, the overall concordance between the resection specimens and CNB was 74% (Table 5), and the kappa coefficient [95% CI] was 0.55 [0.34, 0.75] (*p* < 0.0001).The RS grouping differed between the resection specimens and CNBs for 26% of the patients; however, the RS results were not always higher in either the resection specimen or CNB group. One-step discordance was 26% (13/50), and there was no two-step discordance: 2 of the discrepant cases were in the low-risk group of the resection specimens and the intermediate-risk group of CNBs; 3 and 7 of the discrepant cases were in the intermediate-risk group of resection specimens and the low-risk and high-risk groups of CNBs, respectively; and 1 of the discrepant cases was in the high-risk group of resection specimens and the intermediate-risk group of CNBs.

**Table 5:** Concordance RS, ER, PR and HER-2 value by RT-PCR between paired resection specimen and CNB samples

Resection specimen	CNB										
	RS < 18	RS 18–30	RS ≥ 31	Concordance (%)	RS < 11	RS 11–25	RS ≥ 26	Concordance (%)	Endocrine therapy	Chemoendocrine therapy	Concordance (%)
RS < 18	10	2	0	74.0							
RS 18–30	3	23	7								
RS ≥ 31	0	1	4								
RS < 11					0	1	0	72.0			
RS 11–25					3	21	6				
RS ≥ 26					0	4	15				
Endocrine therapy									9	6	78.0
Chemoendocrine therapy									5	30	
	Negative	Positive						Concordance (%)			
ER status by RT-PCR											
Negative	0	0						100			
Positive	0	50									
PR status by RT-PCR											
Negative	10	4						86			
Positive	3	33									
HER-2 status by RT-PCR											
Negative	50	0	0					100			
Positive	0	0	0								

*ER* oestrogen receptor; *PR* progesterone receptor; *HER-2* human epidermal growth factor receptor-2; *CNB* core needle biopsy

To investigate the low and midrange-risk categories, we used the following TAILORx RS cutoffs: low (< 11), intermediate (11–25), and high (≥ 26) (Table 5). The 21-gene assay demonstrated a low RS in 1 (2%), intermediate RS in 30 (60%) and high RS in 19 (38%) resection specimens (Table 5), while it demonstrated a low RS in 3 (6%), intermediate RS in 26 (52%) and high RS in 21 (42%) CNBs. The concordance between the resection specimens and CNB samples as categorical variables was 72%, and the kappa coefficient [95% CI] was 0.47 [0.25, 0.69] (*p* = 0.001). One-step discordance was 28% (14/50), and there was still no two-step discordance: 1 of the discrepant cases was in the low-risk group of resection specimens and the intermediate-risk group of CNB, 3 and 6 of the discrepant cases were in the intermediate-risk group of resection specimens and the low-risk and high-risk group of CNB, respectively, while 4 of the discrepant cases were in the high-risk group of resection specimens and the intermediate-risk group of CNB.

Andre et al. updated the ASCO Clinical Practice Guidelines for the use of biomarkers to guide adjuvant therapy for early-stage invasive BC in 2019 [42]. The panel recommended that oncologists may offer endocrine therapy or chemoendocrine therapy after the consideration of RS and age. Hence, we grouped the samples as follows: endocrine therapy group (RS < 16, any age; patients older than 50 years with RS of 16–25) and chemoendocrine therapy group (RS ≥ 31, any age; patients 50 years of age or younger with RS of 16–25). The concordance between the resection specimens and CNB samples as categorical variables increased to 78%, and the kappa coefficient [95% CI] was 0.47 [0.20, 0.74] (*p* = 0.003). Six of the discrepant cases were in the endocrine therapy group of resection specimens, while in the chemoendocrine therapy group of CNBs, 5 of the discrepant cases were in the chemoendocrine therapy group of resection specimens and the endocrine therapy group of CNBs.

Strong correlations in ER and PR were observed by RT-PCR (Pearson correlation [95% CI] 0.86 [0.73–0.98], 0.80 [0.65–0.95], respectively), while moderate correlations were observed between the resection specimens and CNBs for HER-2 and Ki-67 (Pearson correlation [95% CI] 0.49 [0.28–0.70], 0.44 [0.22–0.66], respectively). Similar to the RS results, the differences in the ER, PR, HER-2 and Ki-67 results between the resection specimens and CNBs varied in magnitude without any obvious pattern (Fig. 4c–f). There was no discordance in the categorization of ER positivity and HER-2 negativity within the samples; all samples were classified as ER-positive and HER-2-negative (Table 5). By contrast, the PR status of the CNBs differed from that of the resection specimens in 14% of cases: in 3 cases, conversion from a PR-positive resection specimen a PR-negative CNB was observed, and in 4 cases, conversion from a PR-negative resection specimen to a PR-positive CNB was observed (*p* < 0.0001).

## Discussion

Chemotherapy, hormonal therapy, and combinations thereof are three different options for the systemic adjuvant treatment of ER + early BC patients. Treatment recommendations were traditionally based on the patient’s risk of recurrence, and the estimated benefit was balanced against the harm of adverse events resulting from chemotherapy. The management of BC has now entered the era of molecular subtyping with gene expression profiling. The 21-gene assay is the only validated multigene assay for predicting the response to chemotherapy according to the National Comprehensive Cancer Network (NCCN) guidelines [22].

Our study indicated that 24%, 66% and 10% of patients with available resection specimens showed low-, intermediate-, and high-risk RSs, respectively, based on traditional RS cutoffs, while the proportions of patients categorized into the low, intermediate, and high RS risk groups were 2%, 60% and 38%, respectively, according to the TAILORx RS cutoffs [41]. Wu et al. and Wang et al. revealed similar distribution patterns of RSs in Chinese patients [43–45], in which the intermediate-risk patients accounted for over half of the total patients. Notably, the proportions of patients categorized in our study were different from those in the NSABP B-14 trial (low-risk: 51%, intermediate-risk: 22%, high-risk: 27%) [12] and TAILORx trial (low-risk: 17%, intermediate-risk: 69%, high-risk: 14%) [41] or the Surveillance, Epidemiology, and End Results (SEER) program database (low-risk: 58%, intermediate-risk: 35%, high-risk: 7% based on traditional RS cutoffs; low-risk: 14%, intermediate-risk: 60%, high-risk: 26% based on TAILORx RS cutoffs) [46]. We postulated that the discordance might have arisen from the selection of patients by clinicians for enrolment in those clinical trials in whom there was therapeutic equipoise regarding the treatment benefits of the experimental arms (e.g. the extended tamoxifen group in the NSABP B-14 trial and the chemotherapy group in the TAILORx trial). In addition, the overwhelming majority of patients had grade 2 tumours. Previous studies have indicated that the 21-gene RS test may not be necessary in two specific subsets of BCs: (1) grade 1, high-PR, low-Ki-67 cancers (low RS) and (2) grade 3, low-PR, high-Ki-67 cancers (high RS) [47]. When cost and time are considered and the added value of the 21-gene RS test is in question, it may be reasonable to reduce the frequency of 21-gene RS testing. Furthermore, the distribution of RS might differ between Eastern and Western populations, and this potentially inconsistent RS distribution needs further validation.

Several groups have correlated common clinicopathologic data with RS [47–50]. Early validation studies showed that a high tumour grade was significantly correlated with a high-risk RS and the risk of distant recurrence [12, 13]. Institutional studies have demonstrated that tumour grade, T stage, PR status, Ki-67 index and molecular subtypes were associated with a high RS and increased risk of recurrence over 10 years [43, 51–53]. In addition, multiple groups have developed nomograms or models that have correlated pathologic information such as tumour grade, ER status, PR status and Ki-67 with RS [48, 49, 54, 55]. In the PlanB trial, the distribution patterns of RS based on TAILORx RS cutoffs were also found to be significantly associated with tumour grade, PR status and the Ki-67 index [56]. In our study, RS was demonstrated to be significantly associated with T stage, PR status and molecular subtype based on traditional RS cutoffs, while RS was significantly associated with age, PR status and molecular subtype based on TAILORx RS cutoffs (all *p* < 0.05, Table 2). Among patients with PR-negative and luminal B tumours, the proportion of patients with a high-risk RS was significantly higher than that among their counterparts, despite the different definitions of risk categories, similar to previously published data. These facts indicated that routine clinical and pathologic parameters might be helpful in predicting RS.

It is currently recommended that biomarker studies should be performed in all new, recurrent, or metastatic invasive breast carcinomas using IHC and/or FISH methodology [35, 57]. ER, PR and HER-2 status is an important factor in determining whether a patient will benefit from antioestrogen or targeted therapy. Qualitative and quantitative results for ER, PR and HER-2 are included in the 21-gene RS report. With the supplemental reporting of these biomarkers, there has been growing interest in molecular testing by RT-PCR. Conflicting views on the utility of the 21-gene assay as a test for accurately measuring these biomarkers have been published [26, 47, 58, 59]. For example, Badve et al. compared IHC results for ER and PR with those of the 21-gene RT-PCR assay from the Eastern Cooperative Oncology Group (ECOG) 2197 trial [26]. For ER, the concordance between IHC and RT-PCR was 93%, while the concordance was 88% for PR. Park et al. reported 98.9% concordance for ER and 91.3% concordance for PR when IHC and RT-PCR were compared in 265 BC patients [38]. In another study, Kraus et al. showed good concordance of 98.9% for ER and 94.2% for PR status when IHC and RT-PCR were compared [59]. The authors concluded that IHC was slightly more sensitive in determining ER and PR expression. Similar to previously published data, our findings showed a perfect positive concordance of 100% for ER status when IHC and RT-PCR results were compared, whereas the concordance for the PR status was low (80%), which is slightly lower than that reported in other published studies [38, 59]. However, 4 and 6 discordant cases among the resection specimens and CNBs were weakly PR-positive (< 10% of cells), respectively, and the new IHC nuclear staining-positive/negative threshold defined in 2012 (10% prior to 2012 and 1% in 2012) [51] may lower the concordance for the PR status. Indeed, when the cutoff point for PR positivity was set at 10%, the concordance for the PR status was 88% for resection specimens and 92% for CNBs. Prior studies have also shown considerable discordance between IHC/FISH and RT-PCR for HER-2. For example, Park et al. reported good concordance of 99.2% (100% negative agreement and 0% positive agreement) for HER-2 between FISH and RT-PCR [38]. Dvorak et al. demonstrated that the discrepancy rate was 4.1% when comparing the FISH and RT-PCR HER-2 results among 194 cases. Although the overall concordance was 96%, the concordance among HER-2 + cases was only 50%, and there were no patients who were negative by FISH but positive by RT-PCR [60]. In another study, Dabbs et al. reported an overall agreement between IHC/FISH and RT-PCR of 99% for HER-2-negative cases; however, they found an RT-PCR false-negative rate greater than 50% [61]. In our study, the concordance for HER-2 expression was 100% between IHC/FISH and RT-PCR, with 100% negative agreement. The quality of IHC staining can vary depending on many factors, such as the specimen fixation time, reagent batch, instrument, and protocol. At our institution, standard histology protocols as well as automated IHC methods have been used to reduce this variability. One of other hypotheses for the lower concordance for the PR status is the different clones used to generate the PR antibody employed for IHC. Variability can also occur within the RT-PCR procedure, which may result from contamination with non-neoplastic breast tissue, in situ carcinoma, and surrounding inflammatory cells or contamination introduced during the experimental process and may lead to suboptimal results. Discrepancies between the reported results based on IHC, FISH and RT-PCR could cause uncertainty among clinicians and ultimately lead to suboptimal treatments. The routine evaluation of ER, PR and HER-2 by IHC and FISH analysis has been applied in clinical trials, but the results from RT-PCR have not been validated in a prospective trial. Occasionally, HER-2 + cases may be accidentally sent for 21-gene RS testing, and the discrepancies observed in these HER-2 + cases are concerning. These results and others indicate that the assessment of these biomarkers by RT-PCR should be explored.

Several authors have reported the concordance between preoperative CNB and resection specimens for some critical biomarkers used to subgroup and subsequently guide adjuvant treatment decisions in the management of BC. The accuracy of ER, PR and HER-2 status determined by IHC reported in the literature has been found to range from 77.8 to 100% for ER, 69–97.1% for PR, and 60% to 98% for HER-2 [62]. For Ki-67, a few suggestions of cutoffs have been made, and the concordance is controversial [63, 64]. According to the updated cutoffs for ER, PR, HER-2 and Ki-67 [33, 35, 36], the concordance rates between CNB and resection specimens were found to be 100%, 94%, 52% and 82% for ER, PR, HER-2 and Ki-67, respectively, in our study. The concordance for the IHC ER and PR test results was comparable to previously published data [62]. For HER-2 status determined using IHC alone (0, 1 + , and 2 + scores), although the concordance rate (52%) was slightly lower than in other published studies, the negative agreement was 100% for all tumours when HER-2 status was assessed by IHC-FISH as a dichotomous variable (HER-2-negative/HER-2-positive) according to reported recommendations [36]. Proliferation is an essential characteristic of all cancer types, including BC. In the last decade, the proliferation activity of different tumours assessed with IHC detection of the cell-cycle specific antigen Ki-67 has been extensively studied. Many studies have shown that Ki-67 expression is a useful prognostic factor in breast cancer [65]. However, an assessment of cellular proliferation by evaluating Ki-67 expression is not recommended in routine pathological evaluation by either North American Breast Cancer Group and International Ki-67 in Breast Cancer Working Group of the Breast International Group or the ASCO since it is incompletely understood how Ki-67 measurements and thresholds can influence clinical decisions [66]. Ács et al. reported that neoadjuvant chemotherapy (NAC) is more efficient in tumours with at least a 20% Ki-67 labelling index (LI). Both Ki-67 LI and subtype showed a significant association with pathological response. Ki-67 LI was shown to have independent prognostic potential to overall survival (OS), while pathological response did not [67]. The majority of the 13th St Gallen International BC Conference (2013) Expert Panel voted that a threshold of ≥ 20% was clearly indicative of ‘high’ Ki-67 status. According to this updated cutoff, we found a slight difference in high-Ki-67 status of 40% for CNBs and 34% for resection specimens. However, there were a total of 9 discordant cases, which was a rather high rate compared to the discordance rate for PR. Although the concordance rates of ER and PR statuses were high in this study, notable differences for HER-2 and Ki-67 statuses between CNB and resection specimens were found, similar to the published literature [68]. Several explanations have been given for the discordant findings between CNB and resection specimens. Tumour heterogeneity, variation in tissue processing and fixation, and inter- and intra-observer variability are some confounders that have been reported to influence the concordance for HER-2 and Ki-67 [68, 69]. Considering that CNBs only reflect part of a tumour, predominantly the core of the tumour, key information may be missed when tumour heterogeneity is high [17, 70]. The higher discordance rate seen in the PR group compared to the ER group might occur because the PR concentration in the tumour is apparently more variable than the ER concentration [71]. HER-2 expression in a tumour is also believed to vary between different parts of the tumour [72, 73].

As mentioned above, several researchers have reported the concordance between CNBs and resection specimens for some critical biomarkers in the management of BC. The 21-gene RS test has been shown to be prognostic and predictive of chemotherapy benefits in early-stage, HR + , node-negative BC. However, the RS correlation between different specimens has not been intensively examined. Previously, Boolbol et al. demonstrated that a wide distribution of RS results from primary tumours and synchronous nodal metastases showed a modest correlation between 84 paired samples [74], and they recommended that RS testing for ER + BC should continue to be based on the analysis of primary tumours. CNBs may not represent the full biological profile of a tumour in all cases because of sampling error, an insufficient sample size of the CNB, intratumoural heterogeneity, or the menopausal status of patients. However, CNBs may be the only available sample type for molecular testing in patients under neoadjuvant treatment or in cases where there are quality problems in resection specimens caused by improper tissue handling and fixation. The primary goal of the present study was to assess the concordance of the RS results between CNBs and resection specimens. In this study of ER + and HER-2-negative primary breast carcinomas, we observed a modest/strong correlation of continuous gene expression measured according to the RS results and the single-gene analysis results for ER, PR, HER-2 and Ki-67 between paired resection specimens and CNB samples (Fig. 4). In the subsequent analysis, the concordance according to RS group (< 18, 18–30, ≥ 31) was 74%. One-step discordance was 26% (13/50), and there was no two-step discordance. To investigate the low and midrange-risk categories, we also applied low (< 11), intermediate (11–25) and high (≥ 26) cutoffs, and the RS concordance between paired resection specimens and CNBs as categorical variables was 72%. A total of 14 tumours showed one-step discordance; similarly, there was still no two-step discordance. We further grouped the tumours according to the updated ASCO guidelines from the point of view of clinical treatment. The concordance between the resection specimens and CNB samples as categorical variables increased to 78%. These findings suggested that the RS results did not differ between the two kinds of samples, regardless of the different definitions of risk/treatment categories (all *p* < 0.05). In cases where variability exists, there may be actual biological differences. In addition to receptor status, CNBs provide clinicians with foundational information that may or may not correlate with subsequent resection samples [75, 76]. To quantify discordances in RSs between paired samples, the underlying gene expression changes and clinicopathological data that contribute to the discordance should be investigated in detail. In addition, the impact of surrounding non-neoplastic breast tissue or inflammatory cells in resection specimens on RS with the help of mesodissection tools can also be investigated in the future [77].

With respect to the quantitative gene expression of ER, PR, HER-2 and Ki-67, strong/moderate correlations were observed between the resection specimens and CNBs. Similar to the RS results, the differences in ER, PR, HER-2 and Ki-67 between the resection specimens and CNBs varied in magnitude without any obvious patterns. There were no differences in ER or HER-2 status when it was assessed categorically as positive or negative between the resection specimens and CNBs despite differences in continuous gene expression. Notably, there were no paired patient samples that converted from ER-positive to ER-negative or from HER-2-negative to HER-2-positive, which are changes that might prompt the reconsideration of patient treatment. When comparing the PR status between resection specimens and CNBs using the RT-PCR results, a total of 7 tumours were found to be discordant, and 43 were concordant (86%): 3 cases showed conversion from a PR-positive resection specimen to a PR-negative CNB, while 4 cases showed conversion from a PR-negative resection specimen to a PR-positive CNB. As in the above analysis, the IHC PR status of the resection specimen differed from that of the CNB in only 6% of cases: 2 of the discrepant cases showed a weakly PR-positive CNB and a PR-negative resection specimen, while 1 case showed a PR-negative CNB and weakly PR-positive resection specimen. The slightly higher discordance rate seen among the RT-PCR results compared to the IHC results might have occurred because of tumour heterogeneity, in which case IHC is slightly more sensitive in determining PR expression. Notably, PR expression levels according to single-gene analysis were not always higher or lower in the resection specimens. The presence of contamination by surrounding inflammatory cells or non-neoplastic breast tissue may increase or decrease PR expression levels in the resection specimen to some extent.

The strengths of our study include the use of a rigorously, analytically validated quantitative RT-PCR assay rather than IHC, allowing the exploration of the wide dynamic range of the results of the quantitative evaluation of gene expression. These exploratory findings add to the body of literature that has shown the concordance of critical biomarker status in BC between resection specimens and CNBs. The limitations of this study include the inability to fully assess ER and HER-2 expression discordance because patients with ER-negative or HER-2 + primary BC were not included in the study. Although the gene expression results were not the same between the two types of samples, and further investigations are needed, although resection specimens may remain the preferred input material for RS testing, the relative stability of RSs between resection specimens and CNBs would improve the overall decision-making process regarding the administration of complete treatment before the initiation of treatment.

In response to the COVID-19 pandemic, some revised guidelines recommend that physicians use information derived from CNBs to assist in planning for BC care [78]. The intent of this recommendation is to identify candidates for neoadjuvant endocrine therapy for whom surgery may be delayed without significantly compromising long-term outcomes to minimize immunosuppression and coronavirus exposure risk. In addition to receptor status, CNBs provide clinicians with foundational information, including tumour grade and histologic subtype, to inform neoadjuvant treatment decisions. Importantly, our results support guidelines recommending determination of the 21-gene results from CNBs for use in clinical treatment decision making. Use of the CNB is foundational to treatment planning to identify patients with early-stage ER + HER-2- BC and lobular histology, low-intermediate grade tumours, or low-risk genomic assays results for whom neoadjuvant endocrine treatment can be administered when surgery is deferred.

In conclusion, this exploratory study adds meaningful information to the limited body of knowledge regarding the concordance of biomarkers between resection specimens and CNBs. Breast cancer clinicians depend on CNB assessment for histologic features and receptor status to guide neoadjuvant treatment decisions. The data presented in these analyses demonstrate that determination of 21-gene assay results from CNBs is reliable, providing results that closely parallel those obtained from resection specimens. The results of the present study support guideline recommendations for the determination of the RS results from CNBs to assist in neoadjuvant or adjuvant treatment decisions.

## Data Availability

The datasets used and/or analysed during the current study are available from the corresponding author on reasonable request.
